# Active surveillance for carbapenem-resistant *Acinetobacter baumannii* (CRAB) carriage

**DOI:** 10.1128/spectrum.03146-23

**Published:** 2023-11-09

**Authors:** Amir Nutman, Gabrielle D. Levi, Alona Keren-Paz, David Schwartz, Samira Masarwa, Vered Schechner, Yehuda Carmeli

**Affiliations:** 1 National Institute for Antibiotic Resistance and Infection Control, Ministry of Health, Tel Aviv Sourasky Medical Center, Tel Aviv, Israel; 2 Faculty of Medicine, Tel Aviv University, Tel Aviv, Israel; Università Roma Tre, Roma, Italy

**Keywords:** carbapenem-resistant* Acinetobacter baumannii* (CRAB), screening, active surveillance, skin sampling, pre-moistened sponge

## Abstract

Carbapenem-resistant *Acinetobacter baumannii* (CRAB) poses a significant threat in healthcare settings, necessitating robust infection control measures. To identify the most effective sampling method for CRAB carriage detection, we conducted a comparative analysis between skin sampling using a pre-moistened sponge, rectal swabs, and respiratory specimens. Our study encompassed both acute care and post-acute care hospitals. The results revealed that the skin sponge method, when plated on selective chromogenic media, demonstrated the highest sensitivity, exceeding 90% in both settings. Conversely, the commonly employed methods of rectal swabs and respiratory samples combined to exhibit sensitivity levels below 40%. Based on these findings, we recommend implementing the skin sponge method as the preferred approach for CRAB screening in healthcare facilities. This recommendation aims to optimize infection control strategies and mitigate the spread of CRAB in healthcare settings.

**IMPORTANCE:**

Our study’s results provide promising evidence for the incorporation of a high-sensitivity carbapenem-resistant *Acinetobacter baumannii* (CRAB) screening method in healthcare settings. Such an approach could prove beneficial in enhancing infection prevention and control measures, leading to improved patient outcomes and potentially alleviating the burden of CRAB in healthcare systems.

## INTRODUCTION

Carbapenem-resistant *Acinetobacter baumannii* (CRAB) infections present significant challenges in healthcare settings, given their resistance to antibiotics, high mortality rates, and substantial healthcare costs (1). CRAB outbreaks in hospitals are difficult to control and can lead to endemicity, underscoring the urgent need to curtail its spread from both clinical and public health standpoints. In Israel, the incidence of CRAB infections in acute care hospitals (ACH) in 2022 was 3.2 per 10,000 patient-days (unpublished data from the National Center for Infection Control, Israel Ministry of Health).

Detecting carriers is a crucial pillar in controlling the dissemination of resistant organisms in healthcare facilities. Early and accurate detection enables the implementation of targeted infection control measures, such as isolation protocols and enhanced environmental cleaning. However, current guidelines lack specific recommendations regarding the most effective body sites to culture and the optimal laboratory methods for CRAB screening (2 – 4).

In a previous study, we explored the sensitivity of various sampling sites, including the skin, tracheal aspirate, buccal mucosa, and rectum, in detecting CRAB (5). We found that, among 201 patients positive for CRAB in at least one body site, sampling only the skin using a pre-moistened sponge had a 92% sensitivity. All other screening sites had low sensitivity (tracheal aspirate, 49%; buccal mucosa, 63%; and rectum, 47%), and even combinations of two sites (excluding the skin) reached a maximum of 69% sensitivity.

Building upon this earlier investigation, we now present our expanded large-scale experience with CRAB screening in acute care and post-acute care hospitals (PACH).

## MATERIALS AND METHODS

### Study setting and patients

This study included two groups of patients. The first group included patients from PACH who were screened for CRAB as part of a point prevalence survey in 18 PACH in Israel between June 2021 and September 2022. All patients screened had no clinical signs of infection on the day of screening. The second group included patients from 10 ACH who were screened as part of CRAB outbreak investigations conducted between February 2021 and May 2021.

The outcome of interest was screening sensitivity for each body site sampled. Since there is no gold standard for CRAB screening, a patient was defined as a CRAB carrier if a culture from any of the sites sampled was positive. Each CRAB carrier was included in the study only once, i.e. if a patient had a positive sample on successive surveillance, only the first screening was included.

### Specimen collection methods

Sponges pre-moistened with a phosphate buffer (Polywipe; Medical Wire & Equipment, Wiltshire, England) were used to sample the skin by swiping both arms and legs from top to bottom (one sponge for all four limbs). The sponges were held by a gloved hand during the sampling process (https://www.youtube.com/watch?v=NPGJFLW0w7U). Patients were sampled before bathing. After sampling, the sponges were placed in a sterile container with 30 mL of brain-heart infusion (BHI) broth (Hylabs, Rehovot, Israel). Rectal specimens were collected using swabs (Amies agar gel transport swab or eSwab liquid Amies flocked swab; Copan Italia S.P.A., Brescia, Italy). Respiratory specimens were collected by tracheal aspirate from ventilated patients using a suction catheter and placed in a sterile container. Respiratory specimens from non-ventilated patients with a tracheostomy were collected by swabbing the tracheostomy cannula. Skin sponge and tracheal aspirate specimens were collected from ACH patients, while rectal and tracheostomy swabs were not obtained.

### Microbiological methods

All samples were transferred to the National Reference Laboratory and processed on the same day of collection. Skin and rectal specimens were incubated overnight (up to 18 hours) at 37°C in BHI for enrichment. Skin samples were then inoculated onto modified (containing 4.5 µg/mL meropenem) CHROMagar MDR *Acinetobacter* plates (Hylabs, Rehovot, Israel) (6), and rectal samples were inoculated onto CHROMagar mSuperCARBA plates (Hylabs, Rehovot, Israel) and incubated overnight (up to 18 hours) at 37°C (7). Respiratory specimens (tracheal aspirate or tracheostomy swab) were inoculated directly onto modified CHROMagar MDR *Acinetobacter* plates and incubated overnight at 37°C. Suspicious colonies were identified to the species level using *bla*_OXA-51-like_ gene PCR (8). Carbapenem resistance was determined by antibiotic susceptibility testing (AST) for meropenem or by the presence of resistance genes *bla*_oxa-23-like_ or *bla*_oxa-24-like_ (9). AST was performed according to the Clinical and Laboratory Standards Institute M100S guidelines using two different methods: VITEK™-2 (Biomerieux SA, Marcy I'Etoile, France) or disc diffusion (Oxoid, Thermo Fisher Scientific Inc., Waltham, MA, USA).

### Statistical analysis

We calculated the sensitivity for each body site, which refers to the proportion of patients positive for CRAB in that body site alone, among those who tested positive for CRAB in at least one body site. Additionally, we computed the exact 95% CI for these sensitivity values. Analyses were done using Stata version 14.2 (Stata Corporation, College Station, TX, USA).

## RESULTS

We included 487 CRAB carriers, 414 in the PACH group, and 73 in the ACH group. Study participant characteristics are presented in Table 1. Among PACH patients, median age was 71 (IQR 60–80) years, and 54.3% were chronically ventilated. Among ACH patients, median age was 67 (IQR 56–77) years, and 59% were ventilated.

**TABLE 1:** Characteristics of study participants

	Post-acute care hospitals (*N* = 414)		Acute care hospitals (*N* = 73)	
Age, median (IQR), years	71 (60–80)		67 (56–77)	
Male sex, *n* (%)	224 (54.1%)		45 (61.6%)	
Ventilated, *n* (%)	225 (54.3%)		43 (58.9%)	
Ward, *n* (%)	Chronic ventilation	225 (54%)	Intensive care unit (ICU)	15 (21%)
	Skilled nursing	158 (38%)	Internal medicine	15 (21%)
	Sub-acute	18 (4%)	COVID isolation unit	12 (16%)
	Rehabilitation	13 (3%)	Other	31 (42%)

As shown in Table 2, the body site with the highest sensitivity was the skin, with a sensitivity of 95.9% (397/414) in PACH and of 90% (63/70) in ACH (*P* = 0.04). In PACH, respiratory specimens had a low sensitivity of 34.6%: sensitivity was 30.5% (64/210) for tracheal aspirate vs 52.0% (26/50) for tracheostomy swab (*P* = 0.02). The sensitivity of tracheal aspirate specimens was higher in ACH: 76.7%. (33/43).

**TABLE 2:** Carbapenem-resistant *A. baumannii* (CRAB) screening sensitivity among CRAB carriers, by body site

Type of facility	Body site	Number of carriers^*a*^ sampled at body site	Number positive at body site	Sensitivity	95% CI
Post-acute care hospitals	Skin	414	397	95.9%	93.5%–97.6%
	Respiratory (tracheal aspirate or tracheostomy swab)	260	90	34.6%	28.8%–40.7%
	Rectum	383	74	19.3%	15.5%–23.6%
	Respiratory or rectum	401	147	36.7%	31.9%–41.6%
Acute care hospitals	Skin	70	63	90.0%	80.5%–95.9%
	Respiratory (tracheal aspirate)	43	33	76.7%	61.4%–88.2%

^*a*^ A patient was defined as a CRAB carrier if a culture from any of the sites sampled was positive.

## DISCUSSION

In this study, we determined the sensitivity of CRAB screening cultures from different body sites among 487 CRAB carriers in both ACH and PACH settings. We found that culturing the skin using a pre-moistened sponge, which conveniently allows sampling a large skin surface area, had over 90% sensitivity to detect CRAB carriage and over 95% sensitivity in PACH setting.

These findings confirm the results of our previous study, in which skin sampling using similar methods was 92% sensitive for detecting CRAB (5). In a study conducted by Marchaim *et al.* (10), skin swabs were able to detect MDR *A. baumannii* in only 13.5% of patients with recent clinical cultures positive for this pathogen. The differences observed between studies may be attributed to the potential sparsity of CRAB carriage on the skin; thus, it becomes imperative to sample a larger surface area effectively, and this can be achieved by employing a highly absorbent sponge. Consequently, the use of pre-moistened sponges, as opposed to swabs, leads to heightened sensitivity in detecting CRAB carriage. In this study, we found a slightly higher sensitivity of skin sampling in PACH setting (96% vs 90% in ACH). This may be partially explained by difference in bathing practices between settings. Patients in ACH are bathed using antiseptic soap, while those in PACH are bathed using cosmetic soap. However, it should be noted that screening of patients was performed before bathing and that the phosphate buffer in the sponges neutralizes disinfectants.

Respiratory samples are often used to screen for CRAB carriage in ventilated patients (11). We observed variations in the sensitivity of respiratory samples between the two settings, with PACH showing a lower sensitivity (34.6%) compared to ACH (76.7%). This discrepancy can be attributed to differences in the patient populations across these settings. In ACH, a significant proportion of the sampled patients were those hospitalized in the intensive care unit (ICU) and medical step-down units, including COVID-19 dedicated wards. On the other hand, patients sampled in PACH were predominantly those who were chronically ventilated with tracheostomy. These distinctions in patient demographics and clinical conditions likely contributed to the observed variations in sensitivity.

The rectum had a lower sensitivity in our current study than in our previous study: 19.3% vs 47% (5). This may be due to the different populations included in this study, which may have had a lower colonization rate in the feces. Another explanation may be the different microbiological methods used in the two studies. In the current study, we inoculated rectal samples on CHROMagar mSuperCARBA plates, with the purpose of dual screening for CRE and CRAB, while in the previous study, we used a selective media for CRAB, CHROMagar MDR *Acinetobacter* plates. The use of a less selective media may have hindered the detection of CRAB and needs further investigation.

Active surveillance for CRAB is primarily used to control outbreaks, to reduce transmission when incidence is high, or to detect imported cases. Several studies have examined the impact of active surveillance for CRAB. Apisarnthanarak *et al*. (12) evaluated a multifaceted intervention to reduce CRAB in the ICU that included surveillance cultures of tracheal aspirates and rectal swabs on admission and weekly, cohorting and contact isolation of carriers, and enhanced environmental cleaning. The incidence of CRAB acquisition declined by 76%. In a study by Rodríguez-Baño *et al*. (11), a prevention bundle in the ICU that included screening of the rectum, perineum, pharynx, and tracheal aspirate in ventilated patients; cohorting and contact isolation of carriers; and increased attention to hand hygiene and environmental cleaning reduced MDR *A. baumannii* colonization and infection rates by 74% and bacteremia by 84%.

The World Health Organization (WHO) has recognized the significance of CRAB infections and has prioritized development of evidence-based infection prevention and control (IPC) practices for CRAB (3). While WHO guidelines have recommended surveillance as a key component of IPC, there was insufficient evidence to support the recommendation for screening asymptomatic individuals for colonization, and further research was needed to identify optimal microbiological methods for screening. However, recent evidence from two simulation models demonstrates the potential benefits of CRAB screening. One simulation model showed that implementing screening with 90% sensitivity and isolating CRAB carriers could reduce transmission, nosocomial infections, and mortality by approximately 78% when carriage prevalence ranged from 2% to 6%. Moreover, this approach resulted in cost reductions ranging from 22% to 53% (13). In another economic simulation, CRAB screening with a sensitivity of 83% was found to be cost-saving, even when the prevalence of CRAB carriage was low (1%) (14). Thus, the sensitivity of CRAB screening plays a crucial role in determining the cost-effectiveness of active surveillance. Our study findings demonstrate a highly effective method of CRAB screening with over 90% sensitivity. These results align with the simulation models, which indicate that such a screening approach could yield substantial benefits in reducing CRAB transmission, prevent nosocomial infections, and reduce healthcare costs.

The strength of this study is the large number of patients included in 28 different healthcare institutions. The main limitation is the absence of a gold standard for CRAB screening, which resulted in measuring sensitivity at each body site by comparing it to positivity at any body site. It is important to acknowledge that this approach may potentially underestimate the true prevalence of CRAB, which could lead to inflated sensitivity values in our calculations (5).

In conclusion, our study confirms that the skin serves as the primary colonization site for CRAB, underscoring the importance of including it in active surveillance protocols. Conversely, samples from body sites other than the skin, either alone or in combination, demonstrated low sensitivity, rendering them unsuitable for ruling out CRAB carriage. Based on our findings, we recommend that the primary site for CRAB screening should be the skin using a highly absorbent sponge to effectively detect carriage. If screening does not encompass the skin, a negative test result should be accompanied by a comment indicating that “CRAB carriage cannot be ruled out without skin sampling.”.

While current guidelines may lack sufficient evidence to firmly recommend CRAB screening, our study’s results provide promising evidence for the incorporation of a high-sensitivity CRAB screening method in healthcare settings. Such an approach could prove beneficial in enhancing IPC measures, leading to improved patient outcomes and potentially alleviating the burden of CRAB in healthcare systems.
